# Notes on the Vaccination of Guinea-pigs with B. Perfringens

**Published:** 1917-12

**Authors:** 


***Notes on the Vaccination of Guinea-pigs with B. perfringens.*** By Muriel ROBERTSON, Lister Institute of Preventive Medicine. Abs. from the *Lancet*, Sept. 16, 1916.

R. gives in detail the results of vaccination experiments on a large number of guinea-pigs (over a hundred) to determine the resistence created in animals by previous vaccination with killed or attenuated cultures of B. perfringens (aerogenes capsulatus).

For the purpose of the experiments R. found that from 60 to 100 million living organisms of from a 24 to 48-hour broth culture of a virulent B. perfringens might be taken as a lethal dose for a guinea-pig of 250 to 300 grams. Saline solutions of the bacilli grown on nutrient glucose agar were observed to be of relatively very slight infectivity. The vaccine used was usually a saline emulsion killed by heat. Whatever the method of procedure the results were for the most part the same. Out of 62 guinea-pigs vaccinated or treated in any way preventively, only 7 (11.2 o/o) survived a lethal dose of living bacilli. Out of 30 control guinea-pigs, 4 survived the lethal dose, that is, 13.3 o/o.

R. concludes "that previous vaccination with killed or attenuated cultures of B. perfringens does not cause any appreciable raising of the resistence of guinea-pigs against a subsequent lethal dose of living bacilli. Recovery from a previous infection with the organism does not prevent a repetition of the illness upon re-inoculation with living bacilli, nor does it apparently in any way alter the symptoms or influence the course of the disease".

